# Phellodendron chinense Schneid: A novel yellow-emitting luminescent material for white light-emitting diodes

**DOI:** 10.1038/s41598-017-09291-1

**Published:** 2017-08-21

**Authors:** Pin-Chun Lin, Kuei-Ting Hsu, Ming-Hsiu Shiu, Wei-Ren Liu

**Affiliations:** 1 Department of Chemical Engineering, Chung Yuan Christian University, Chungli, 32023 Taiwan; 2 Department of Chemical Engineering, Army Academy, Longdong Rd., Chungli, 32023 Taiwan; 3 Everlight Electronics Co., LTD, No. 25, Lane 76, Sec, 3, Chung Yang Rd., Tucheng Taipei City, 23671 Taiwan

## Abstract

To facilitate the next generation of environmental material for white light emitting diodes, the discovery of natural luminesce is essential. In this study, we disclose a rare-earth free and yellow-emission phosphor, Phellodendron, which could be both excited by near ultraviolet light and blue light. The new yellow phosphor is obtained by extraction of Phellodendron chinense Schneid. The emission wavelength, full width at half maximum and CIE coordinates of extracted Phellodendron are 540 nm, 120 nm and (0.41, 0.55), respectively. The corresponding luminescent properties of Phellodendron are characterized by PL, PLE, reflection spectra, FITR and decay lifetime. Surprising thing is luminous intensity of Phellodendron phosphors excited at 380 nm was stronger than YAG:Ce phosphor by more than 139%. In addition, we firstly introduce the yellow phosphor in white LED fabrication by combining blue chip and Y_3_Al_5_O_12_:Ce^3+^ phosphor, to create warm white. For comparison, red-emission CaAlSiN_3_:Eu^2+^ phosphors are also introduced for LED package tests. The results demonstrate that Phellodendron is a potential candidate for white LED applications.

## Introduction

Nowadays, white light emitting diodes (W-LEDs) have provided remarkable advances in lighting technologies and displays^1^. Commercially-available W-LEDs are fabricated by combining either a blue-emitting InGaN-based LED chip covered by a yellow-emitting phosphor Y_3_Al_5_O_12_:Ce^3+^ (YAG)^2–5^ or n-UV LED chip with coupling red, green and blue tricolor phosphors^6–9^. A large number of luminescent materials used for LED applications are based on rare-earth ions doping technology^1, 10–12^. Given a history of ecological concerns about pollution from rare earth mines, particularly in China, there are growing social and environmental concerns about the growth of the mining and mineral processing in this sector^13–15^. Apart from rare-earth ions doping, nature of narrow absorption for YAG limit its application for n-UV LED application. Advent of technological advancement and economic growth have put our environment at great risk which has led researchers and scientists to develop technology that generates less negative environmental impact. We need to find some substitute for reducing the usage of rare-earth ions, preferably.

Phellodendron is a deciduous tree in the family Rutaceae that is native to east and northeast Asia^16, 17^. Phellodendron bark mainly composed of berberine and palmatine^18–20^, which have been vastly used in Chinese traditional medicine^21–25^ for various symptomatic treatment and pharmacological activities, such as inflammation^26, 27^, anti-diarrhoea^19, 25^, antitumor^28–31^, antiviral^18, 32, 33^, and pneumonia^34^. Aside from its importance in traditional and modern pharmaceuticals, Phellodendron bark has been one of the major sources of yellow dye in Asia for fibers and paper materials not only for its color but also for its insect repellency^35–39^. Berberine and palmatine have received much attention in the field of medicine, dyes and dyeing since it is one of the few cationic colorants of the natural plant dyes, and it has high alkaloids^40, 41^. Furthermore, Zhang *et al*. found the yellow varnish on the metallic foil of a 19–20th century Tibetan altar, the presence of berberine and possible palmatine as well, by using HPLC-MS analysis^42^. Its major chromophoric substance is known to be berberine and palmatine which dyes fibers into yellow color from Phellodendron bark. To the best of our knowledge, there is no study on both the luminescence properties and LED applications of yellow material from Phellodendron bark. In this study, we firstly demonstrate that Phellodendron phosphor by extracting natural Phellodendron bark with ether and composition and luminescence of Phellodendron phosphor were investigated. In addition, in order to reduce usage of rare-earth ions, we combine commercial phosphor and natural Phellodendron phosphor by extracting.

In this study, we found a natural yellow material by extracting Phellodendron chinense Schneid. This yellow-emission phosphor is believed to replace rare earth ion-doped phosphors and reduce their usage in LED applications. Phellodendron phosphor could be pumped by near-UV light or blue light because of its nature of wide absorption band. We construct a series of LED package data by using YAG, CaAlSiN_3_:Eu^2+^ and Phellodendron phosphor for comparison. The proposed approaches may be alternative combination for LED applications in the future.

## Results and Discussion

**Figure 1:**
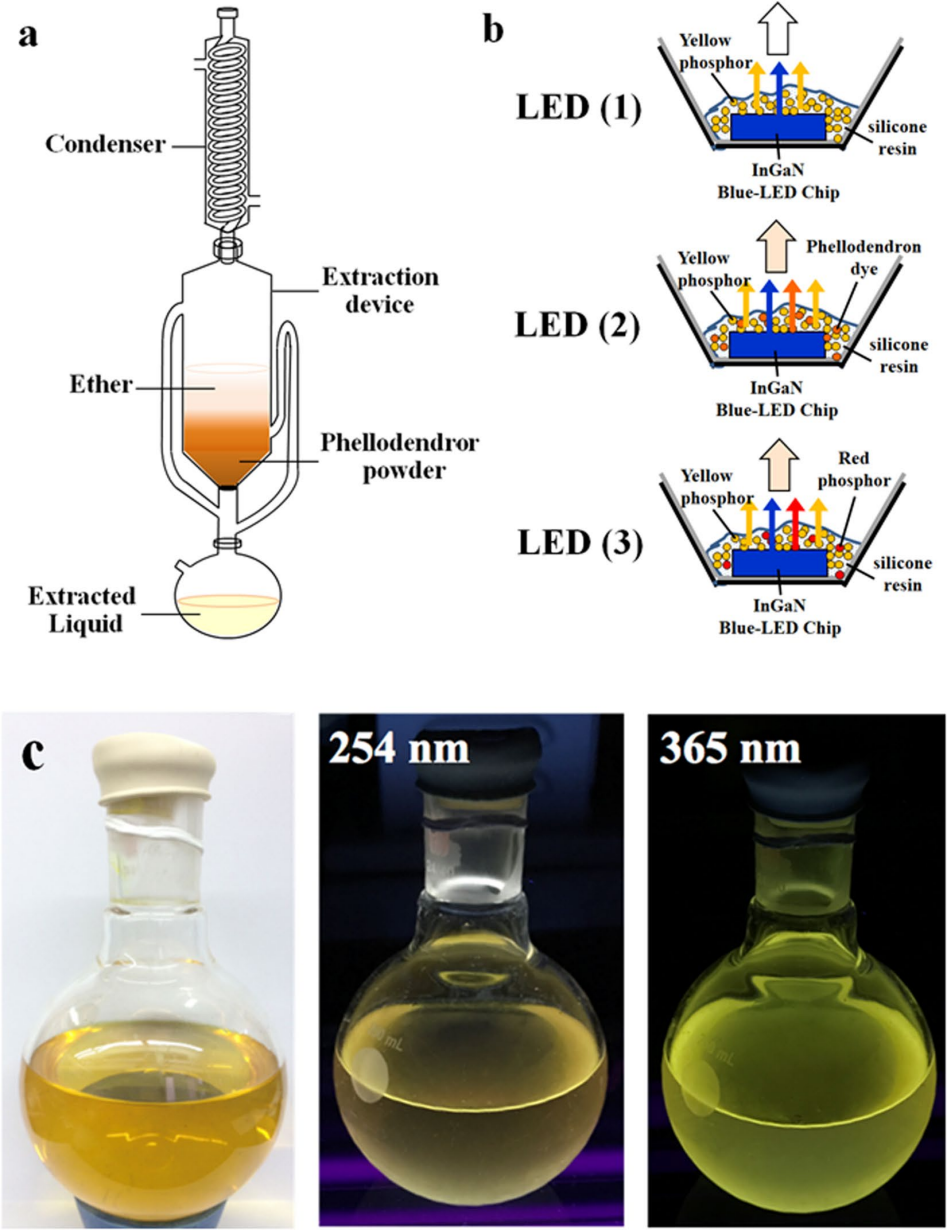
(**a**) Schematic of the extracted device for Phellodendron phosphor; (**b**) Illustration of w-LEDs packaged for LEDs; (**c**) Photograph of Phellodendron phosphor excited at 254 nm and 365 nm in UV box.

**Figure 2:**
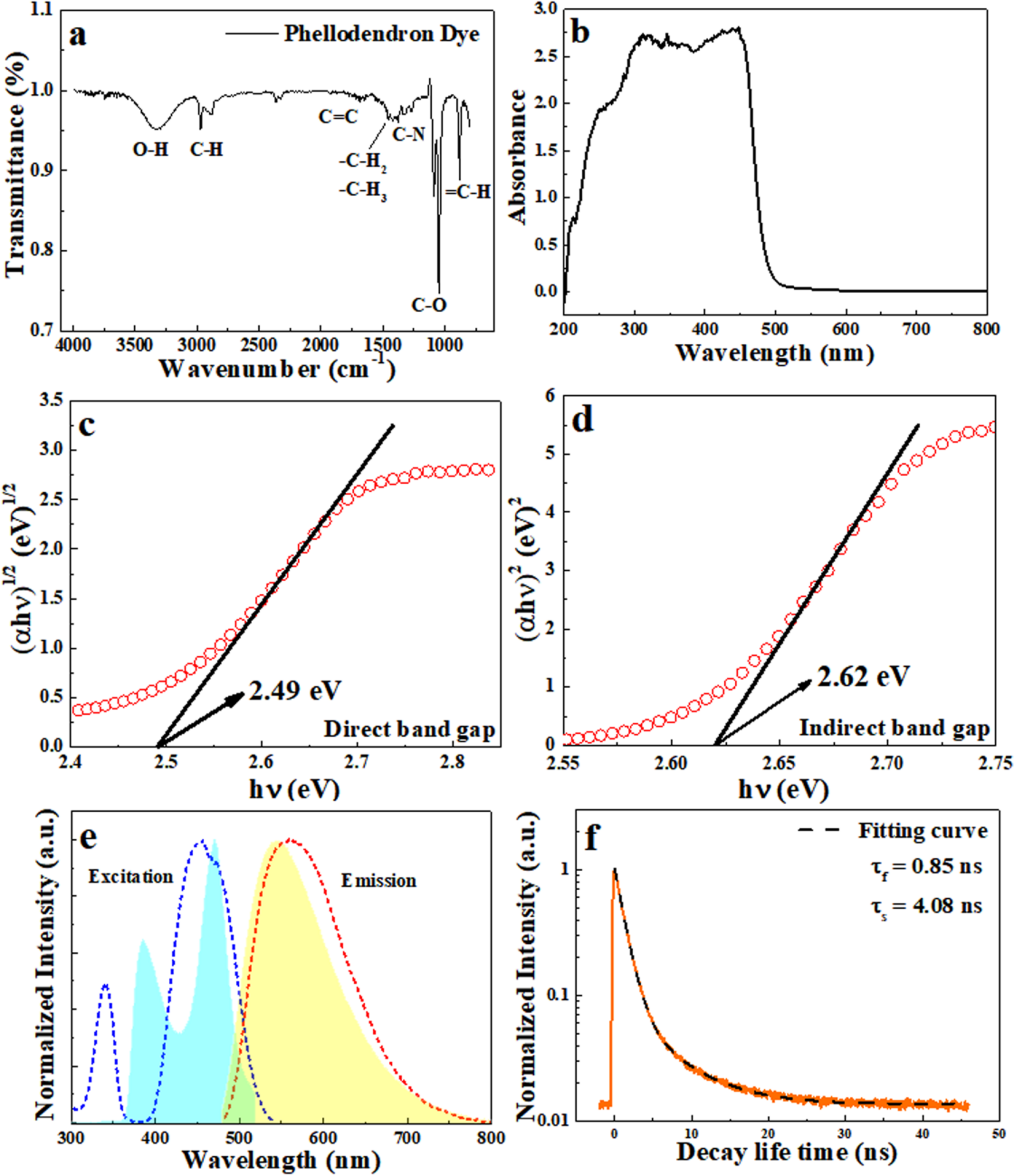
(**a**) FT-IR spectrum of Phellodendron phosphor; (**b**) UV visible absorption spectrum of Phellodendron phosphor; (**c**) Direct band gap estimation of Phellodendron phosphor; (**d**) Indirect band gap estimation of Phellodendron phosphor; (**e**) Relative photoluminescence excitation and emission spectra of Phellodendron phosphor (filled area) and YAG:Ce (dotted curves); (**f**) The PL decay spectrum of Phellodendron phosphor monitored at the emission peak (orange line). The dash line shown in (**f**) is a fitting result according to Eq. (2).

The photo image of Phellodendron phosphor is shown in Fig. 1c which was extracted by using diethyl ether and collected and evaporated in rotary evaporators to obtain the diethyl ether extraction. The daylight color shown in Fig. 1c was amber yellow. In UV box, Phellodendron phosphor displayed mustard yellow and canary yellow under 254 nm and 365 nm excitation, respectively. Since the alkaloids in Phellodendron chinense Schneid were high polar, methanol-d_4_ was used as a solvent to ensure that all the extract can be dissolved and avoid the inference of phenolic hydroxyl signals for NMR measurement^18^. The ^1^H NMR spectrum of Phellodendron phosphor is well documented in methanol-d_4_ in Fig. S2 and Table S1. The analysis of the NMR spectrum of Phellodendron phosphor revealed the extracts is Berberine^18^. FT-IR spectroscopy was used to analyze to the functional groups in Phellodendron phosphor, as shown in Fig. 2a. The FT-IR spectrum revealed the presence of = C-H (880–995 cm^−1^), C-O (1040–1100 cm^−1^), C-N (1250–1271 cm^−1^), CH_2_ & CH_3_ (1350–1470 cm^−1^), C=C (1630–1680 cm^−1^), C-H (2850–3000 cm^−1^), -OH (3200–3550 cm^−1^) in Phellodendron phosphor. The result of FI-TR spectroscopy was consistent with the result of NMR.

The optical property of Phellodendron phosphor was analyzed using UV visible spectroscopy and the corresponding spectrum is shown in Fig. 2b. The broad absorption peak was from 200 nm to 500 nm. The absorption coefficient α in many amorphous semiconductors shows an exponential dependence on photon energy usually obeying the empirical relation. The band gap of Phellodendron phosphor (*E*_*g*_) was determined by using Tauc’s plot^43–46^.

1 $${\boldsymbol{\alpha }}h\upsilon =\beta {(h\upsilon -{E}_{g})}^{n}$$

where *h*—Planck’s constant, *υ*—frequency of vibration, *α*—absorption coefficient, *E*_*g*_—band gap, *β*—proportional constant. For allowed direct transitions, *n *= 1/2, and for allowed indirect transitions, *n *= 2. From results of Fig. 2(c) and (d), Phellodendron phosphor showed both direct band gap and indirect band gap were determined to be 2.49 eV and 2.62 eV, respectively.

Normalized photoluminescence excitation and photoluminescence emission spectra of Phellodendron phosphor and YAG:Ce are displayed in Fig. 2e. Phellodendron phosphor exhibits a nature of broad absorption band from 360 nm to 500 nm, indicating that it can be effectively applied in general lighting for UV, nUV and blue-pumping LED devices. The emission spectra of Phellodendron phosphor and YAG:Ce excited at 460 nm display the broad yellow-emitting peak at 543 nm and 560 nm, respectively. YAG:Ce, however, could only be excited at 460 nm according to the sharp absorption band at ~460 nm. Even though, the luminous intensity of Phellodendron phosphor is 3% of that of YAG:Ce phosphor at 460 nm excitation. Nevertheless, when Phellodendron phosphors were excited at 380 nm, the luminous intensity of Phellodendron phosphor is more than 139% than that of YAG:Ce phosphor. Phellodendron phosphor can be applied both nUV and blue chip for LED devices following to the above result. Fig. S3a shows PL/PLE spectra of commercial red-emission phosphor-CaAlSiN_3_:Eu^2+^. The emission spectra of CaAlSiN_3_:Eu^2+^ phosphor is excited at 460 nm displays red-emitting peak at 620 nm.

To better explain information about the microenvironment surrounding the excited probe molecule, we recorded the time-resolved PL (TRPL) decay measurements of Phellodendron phosphor, as shown in Fig. 2f. It provides a complementary technique to study the PL mechanism of Phellodendron phosphor, using the kinetics of electron-hole recombination as a probe. The orange line in Fig. 2f displays the PL decay profile of the Phellodendron phosphor under excitation of the 260 nm laser. The PL profile can be well fitted by a sum of exponential expressions (equation 2) and the average lifetimes (*τ*) for bi-exponential decay of fluorescence were calculated from the decay times and pre-exponential factors, using the following equation (equation 3) as previous work^47^:

2 $${\rm{I}}({\rm{t}})=\sum _{i}^{n}{\alpha }_{i}\exp (-t/{\tau }_{i})$$

3 $$\tau =\frac{{\alpha }_{i}{\tau }_{i}^{2}}{{\alpha }_{i}{\tau }_{i}}$$

where *α*_*i*_ is amplitudes and *τ*_*i*_ is the decay lifetimes. The behavior of bi-exponential decay of Phellodendron phosphor indicate that there were a fast component *τ*_*f*_ (0.85 ns) and a slow component *τ*_*s*_ (4.08 ns). The phenomenon is similar with previous studies, such as carbon quantum dots and luminesce dyes^47^. According to the above equations, the decay curve in Fig. 2f gives a average time constant of *τ* = 2.47 ns.

**Figure 3:**
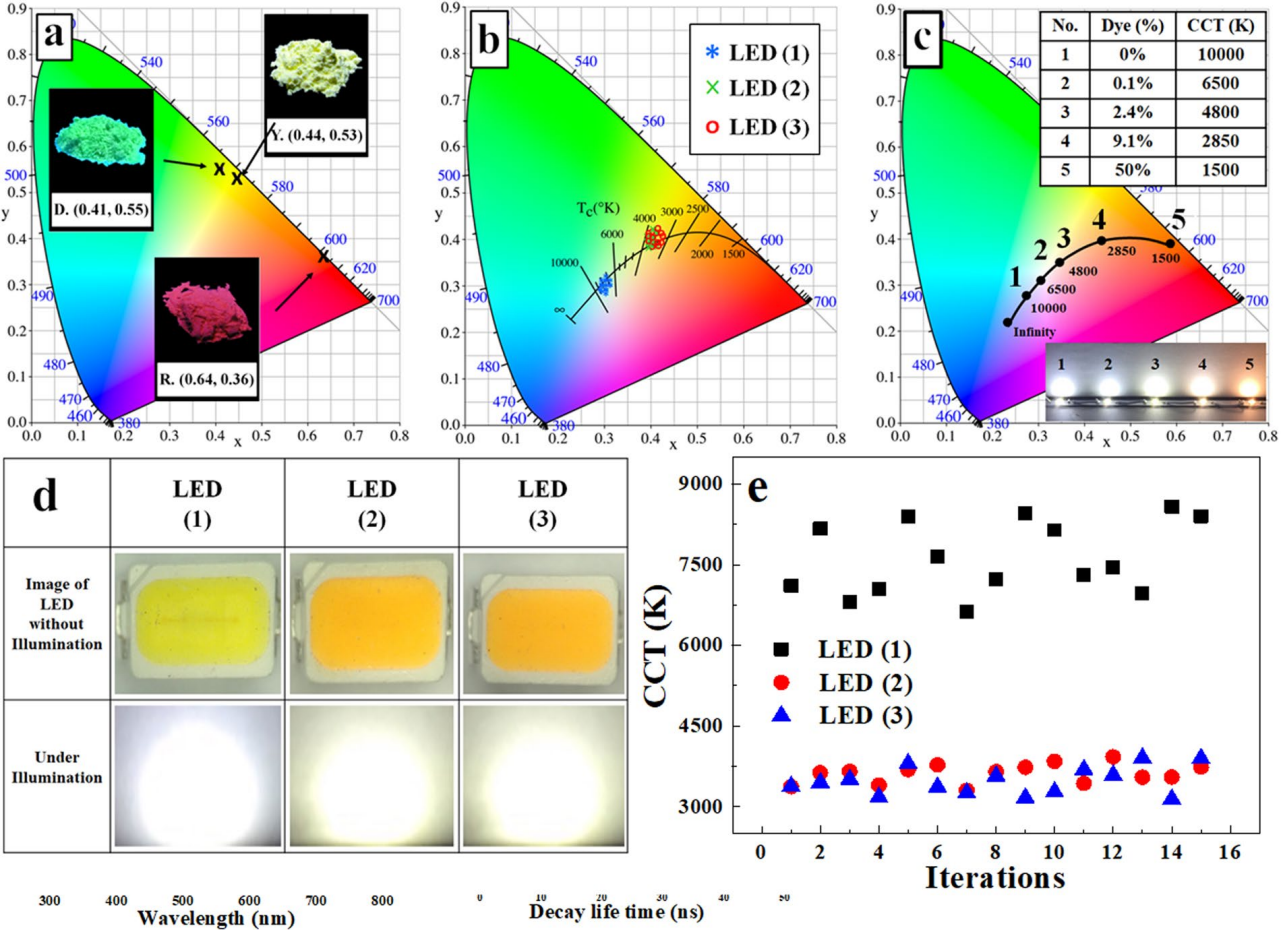
(**a**) CIE chromaticity diagram of Phellodendron phosphor (D), YAG phosphor (Y) and CaAlSiN_3_:Eu^2+^ phosphor (R). Insets: the images of samples excited with blue light in box; (**b**) CIE chromaticity diagram of w-LEDs coupling the 460 nm blue chip with LEDs (*: LED (1), ×: LED (2), o- LED (3)); (**c**) CIE chromaticity diagram of LEDs with different content (0, 0.1, 2.4, 9.1, 50%) Phellodendron phosphor; (**d**) Photographs of the LED lamp packages under 30 mA forward bias currents; (**e**) Color correlated temperature (CCT) of LED (1), LED (2) and LED (3).

The Commission Internationale de L’Eclairage (CIE) color diagram of above Phellodendron phosphor (D), YAG phosphor (Y) and CaAlSiN_3_:Eu^2+^ phosphor (R) are shown in Fig. 3a. The CIE coordinates of three samples are marked in CIE 1931 chromaticity diagram beyond D (0.41, 0.55), Y (0.44, 0.53), and R (0.64, 0.36), respectively. The images of samples are excited under 365 nm in UV box. Furthermore, in order to demonstrate the promising application of Phellodendron phosphor in solid-state lighting. We package LED by combing Phellodendron phosphor and commercial YAG:Ce phosphor. Due to the high CCT (over>5000 K) of commercial W-LED (blue LED + YAG:Ce), it produces a cold bluish white. Owing to the continuous advances in phosphor technologies, tunable CCT from 2700–6500 K can be achieved^48^. W-LEDs are constructed by combing Phellodendron phosphor and the commercial YAG:Ce phosphor with an InGaN-based blue LED chip in Fig. 3d. The commercial W-LED (Blue LED + YAG:Ce, namely LED(1)) was compared with the commercial W-LED (Blue LED, YAG:Ce, and Phellodendron phosphor, LED(2)) and the commercial W-LED (Blue LED, YAG:Ce, and CaAlSiN_3_:Eu^2+^, LED(3)). As a result, CIE coordinates of W-LEDs are changed from high CCT to low CCT after adding Phellodendron phosphor or CaAlSiN_3_:Eu^2+^ phosphor in W-LEDs. Photographs of the W-LED lamp packages under 30 mA forward bias currents are shown in Fig. 3b. After adding Phellodendron phosphor or CaAlSiN_3_:Eu^2+^ phosphor into phosphor paste, the color temperature of W-LEDs changed from cold color temperature to warm color temperature. Fig. S2c exhibits the CIE chromaticity diagram of enlarge of Fig. 3b with Planckian Locus line. The CIE chromaticity coordinates are shown in Table S2.

Figure 3e shows the CCTs of LED (1), LED (2) and LED (3) with 15 iterations each and the summary are shown in Fig. S3c and Table S3. The range of LED (2) and LED (3) were from 3000 K to 4000 K, which are lower than that of LED (1). The average CCT of LED (1), LED (2) and LED (3) are 7623 K, 3615.7 K and 3477.6 K, respectively. It indicates that the CCT of W-LEDs could be easily controlled through the addition of Phellodendron phosphor or CaAlSiN_3_:Eu^2+^ phosphor. Based on the results, the CCT of LED (2) was similar to that of LED (3). Thus, the Phellodendron phosphor could replace the commercial red phosphor CaAlSiN_3_:Eu^2+^.

In addition, we packaged LEDs with different content (0, 0.1, 2.4, 9.1, 50 wt.%) of Phellodendron phosphor shown in Fig. 3c. More Phellodendron phosphor we added, the color of emitting light is much warmer from 10000 K to 1500 K under 30 mA forward bias currents. In other words, we can easily control CCTs of LEDs through the Phellodendron phosphor addition for different applications. For luminous efficacy, the white LEDs driven of LED(1), LED(2) and LED(3) at a D.C. current of 30 mA exhibited about 61.6, 48.6 and 58.8 lm/W, respectively. By characterizing the luminesce properties of Phellodendron phosphor, we revealed that the yellow-emission peak at 543 nm and it could partially replace rare-earth elements and reduce their use in LED applications.

## Conclusions

In this study, we first report luminescent properties of phosphor by extracting natural Phellodendron bark with ether. Phellodendron phosphor can be made into powder and be suspended in epoxy resin to form phosphor resin. Nuclear magnetic resonance (NMR), Photoluminescence/ photoluminescence excitation (PL/PLE), as well as CIE coordinates are characterized. The results indicated that phosphor gives an intense yellow emission peak at 540 nm under optimal excitation wavelength of 460 nm. The absorption band of phosphor was in the range of 350–525 nm, which could be excited by either n-UV chips or blue chips. The amount of phosphor contain can thus be adjusted to optimize the color intensity efficiency, CIE coordinate, correlated color temperature (CCT) and color-rendering index (CRI) of the light emitting diode (LED). Furthermore, this study helps with selection of the appropriate phosphorus content so on to optimize CIE and CCT performance.

## Methods

### Materials synthesis

In this study, Phellodendron chinense powder (40 g) was extracted by using 1 L diethyl ether in nitrogen atmosphere for 2 hours under reflux. Then, the supernatant was collected and evaporated to dry in rotary evaporators to obtain the diethyl ether extract, named Phellodendron phosphor. Figure 1(a) shows the schematic of the extracted device for Phellodendron phosphor. Phellodendron chinense powder was purchased from the Sheng Chang pharmaceutical Co., Ltd. Technology. Diethyl ether (HCOOH, >98%) was purchased from Sigma-Aldrich (USA). For the W-LED fabrication, 0.1 g phosphor were mixed with 0.1 g thermal-curable silicone resin (OE-6636A) under vigorous stirring. Subsequently, silicone resin (OE-6551B, 0.1 g) was added to form a paste. The procedure of LED packing by LED dispenser is shown in Fig. S1. W-LEDs were fabricated by dispensing the phosphor containing paste onto the InGaN-based blue-LED chip (ES-CEBLV10R). In this paper, W-LEDs device we used could be divided into three types: LED (1): Blue chip (455~460 nm)  + YAG (0.1 g, UC-521B); LED (2): Blue chip (455~460 nm)  + YAG phosphor (0.1 g, UC-521B) + Phellodendron phosphor (0.01 g) and LED (3): Blue chip (455~460 nm) + YAG phosphor (0.1 g, UC-521B) + CaAlSiN_3_:Eu^2+^ phosphor (0.00475 g, CN-NR630). Figure 1(b) exhibits the illustration of w-LEDs packaged for LEDs and all conditions were packaged with 15 iterations each.

### Characterization methods

The photoluminescence (PL) and photoluminescence excitation (PLE) spectra were measured using Horiba Jobin-Yvon FluoroMax-4 spectrometer equipped with a 450 W Xenon lamp as the excitation source. All the spectra were measured with a scan rate of 150 nm min^−1^. ^1^H NMR spectra were measured in methanol-d_4_ ( > 99.8%, Sigma-Aldrich) with a Bruker Avance 400 MHz spectrometer, using TMS as an internal standard. For each sample, 100 scans were recorded with the following parameters: 0.183 Hz/point with spectra width of 6009 Hz. The pulse width and relaxation delay are 4 s and 1.5 s, respectively. For quantitative analysis, peak area was integrated for each peak selected manually. Fourier transform infrared spectroscopy (FT-IR) spectra were collected using a Bruker Tensor 27 FT-IR spectrophotometer at a resolution of 4 cm^−1^. The optical absorption spectra of the samples were carried out with an UV–VIS-NIR spectrophotometer (Hitachi U-3900, Japan) in the range of 200–800 nm. Commission International de I’Eclairage (CIE) chromaticity coordinates of the samples were measured using a Laiko DT-101 color analyzer equipped with a CCD detector (Laiko Co., Tokyo, Japan). A pulsed laser with a wavelength of 260 nm, a repetition frequency of 20 MHz, and a duration of 250 fs was used as the excitation source for time-resolved PL studies. The collected luminescence was dispersed by a 0.75 m spectrometer and detected with the photomultiplier tube. Time-resolved PL were performed using the technique of time-correlated single-photon counting (TCSPC). The steady-state and time-resolved PL was measured at room temperature. The time resolution for the time-resolved PL instrument is pico-second.

## Electronic supplementary material

Supporting information
