# Supplementary material for: Phellodendron chinense Schneid: A novel yellow-emitting luminescent material for white light-emitting diodes
Source: Sci Rep. 2017 Aug 21;7:9009. doi: 10.1038/s41598-017-09291-1 (PMC5567165; doi:10.1038/s41598-017-09291-1)
Supplement: Supplementary file 1 — Supporting information [file 41598_2017_9291_MOESM1_ESM.pdf]

Supporting Information

Phellodendron Chinense Schneid: A novel yellow-emitting luminescent material for white light-emitting diodes

Pin-Chun Lin[a], Kuei-Ting Hsu[b,*], Ming-Hsiu Shiu[c] and Wei-Ren Liu[*]

[a] *Department of Chemical Engineering, Chung Yuan Christian University, Chungli, 32023, Taiwan, R.O.C.*

[b] *Department of Chemical Engineering, Army Academy, Chungli 32023, Taiwan, R.O.C.*

[c] *Institute of Organic and Polymeric Materials, National Taipei University of Technology, No.1, Section3, ZhongxiaoE.Rd., Da'an Dist., Taipei City106, Taiwan*

a **b**

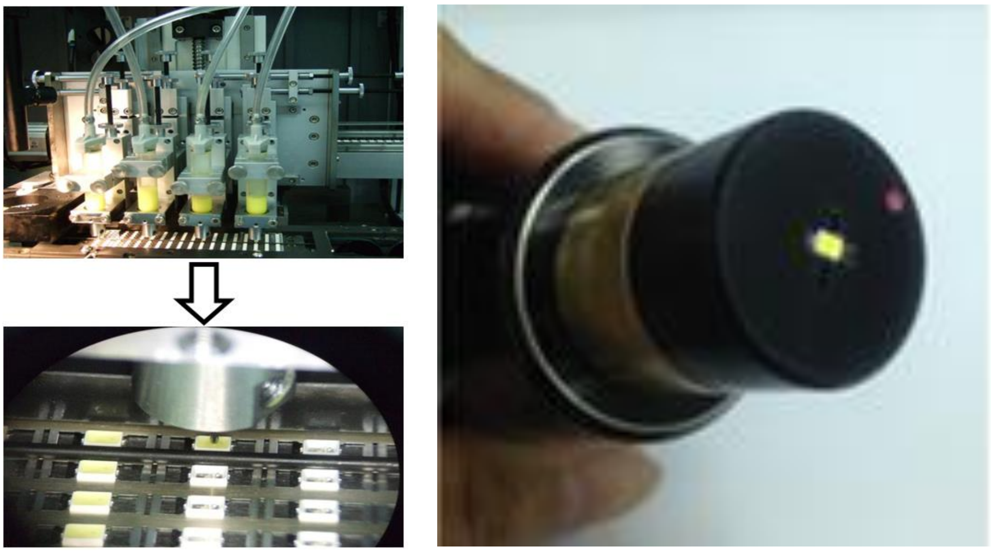

Figure S1 (a) Schematic diagram of LED dispenser. (b) Luminous intensity measurement adapters for LEDs (Instrument Systems LED-4xx).

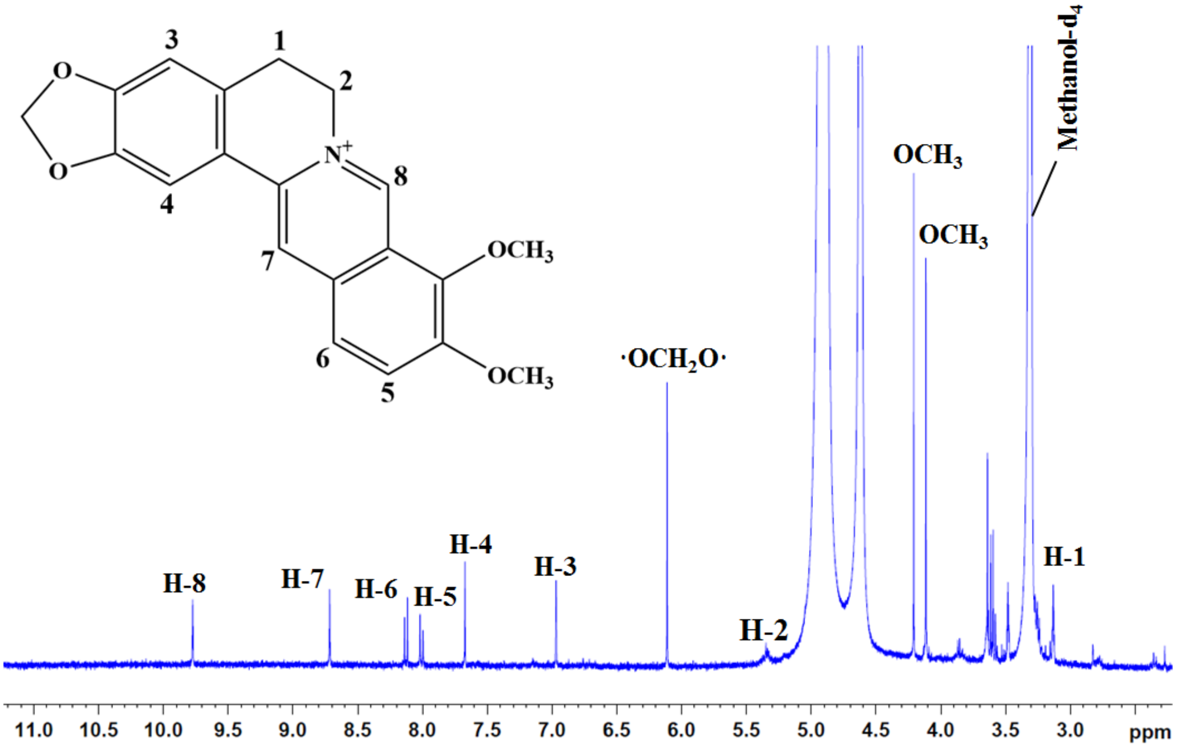

Figure S2 The ^{1}H NMR spectra of Phellodendron phosphor in methanol-d$_4$.

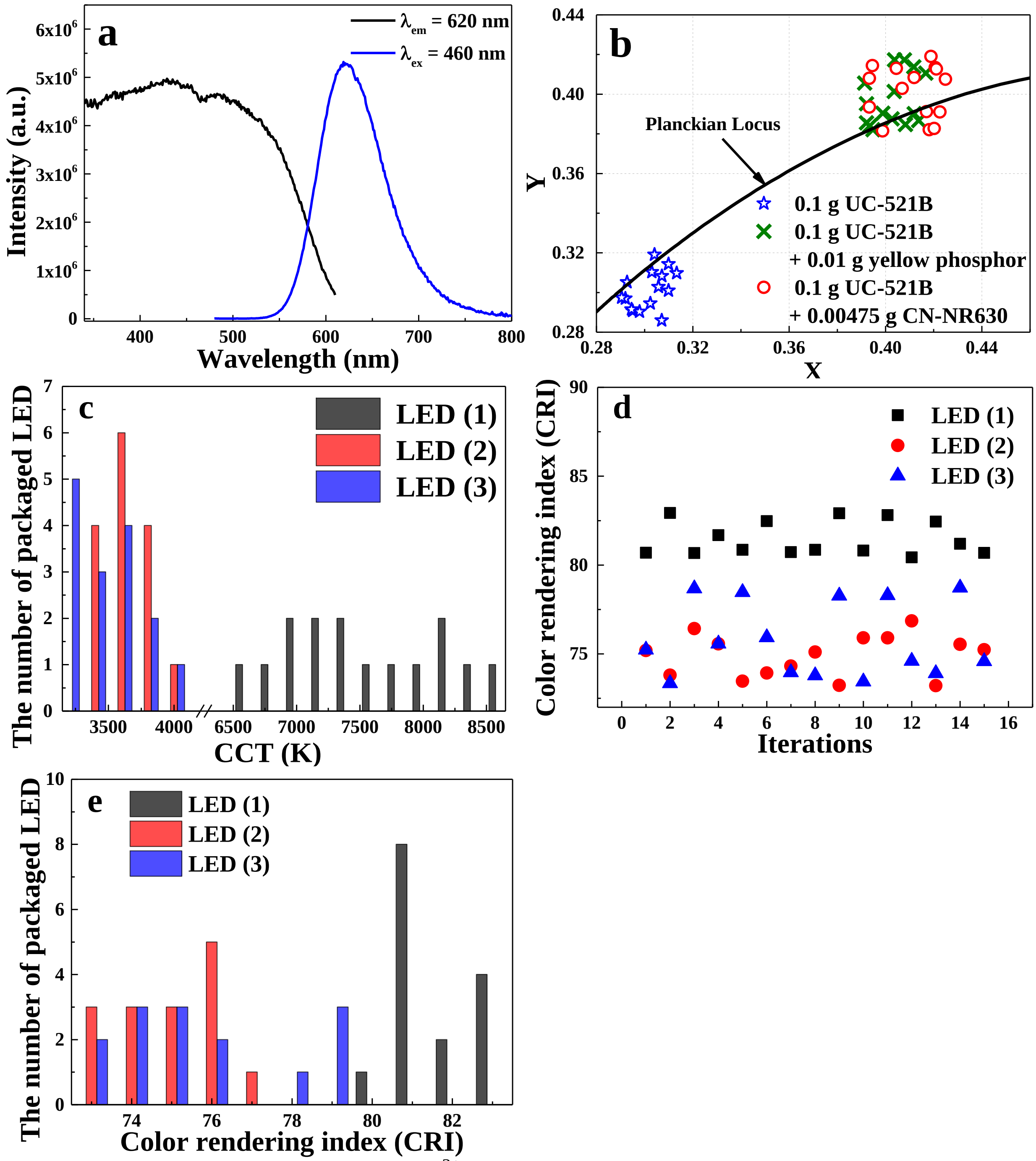

Figure S3. (a) PL/PLE of $CaSiAlN_3:Eu^{2+}$ phosphor excited at 460 nm and monitored at 620 nm, (b) CIE chromaticity diagram of enlarge of fig. 4 (b) with Planchian Locus line. The CIE chromaticity coordinates are shown in Table S1, (c) Summary of correlated color temperature in fig. 4 (d) for LEDs, (d) Color rendering index (CRI) of LED (1), LED (2) and LED (3) and (e) Summary of color rendering index in fig. S2 (d) for LEDs.

Table S1 ^{1}H NMR (400 MHz) spectral data of Phellodendron phosphor.

Position	δ_H (*J* in Hz)
1	2.83
2	5.34
3	6.97
4	7.67
5a	8.00
5b	8.02
6a	8.12
6b	8.14
7	8.72
8	9.77
-OCH_3	4.11 & 4.21
•OCH_2O•	6.11

Table S2 The CIE chromaticity coordinates of LED (1), LED (2) and LED (3). Each condition was packaged with 15 LEDs.

CIE	LED (1)		LED (2)		LED (3)	
Num.	x	y	x	y	x	y
1	0.3041	0.3191	0.4119	0.3902	0.4207	0.4134
2	0.2927	0.3052	0.4036	0.4014	0.4189	0.419
3	0.3099	0.3143	0.3989	0.3904	0.4119	0.4084
4	0.3072	0.3083	0.4083	0.3847	0.4182	0.3821
5	0.295	0.2907	0.3948	0.3821	0.3933	0.3935
6	0.3024	0.2945	0.3921	0.3856	0.4211	0.4127
7	0.3133	0.3098	0.4138	0.3867	0.4249	0.4075
8	0.3058	0.3028	0.4078	0.4173	0.4069	0.4029
9	0.292	0.297	0.3935	0.3841	0.4226	0.391
10	0.2979	0.2903	0.3921	0.3952	0.417	0.3912
11	0.303	0.3103	0.4167	0.4106	0.4045	0.413
12	0.3071	0.2859	0.3913	0.4056	0.3988	0.3815
13	0.3099	0.301	0.4027	0.3876	0.3946	0.4144
14	0.2905	0.2974	0.4117	0.4138	0.4202	0.3827
15	0.2946	0.2915	0.4037	0.4173	0.3933	0.408
Avg.	0.3017	0.3012	0.4029	0.3968	0.4111	0.4014

Table S3 The color correlated temperature (CCT) of LED (1), LED (2) and LED (3). Each condition was packaged with 15 LEDs.

Num.	LED (1) (K)	LED (2) (K)	LED (3) (K)
1	7108.5	3370.9	3375.9
2	8171.1	3629.6	3448.9
3	6800.6	3653.1	3508.8
4	7051.1	3401.8	3174.7
5	8393.6	3685.9	3803.4
6	7653	3776.2	3363.4
7	6630.6	3303.1	3254.7
8	7232.2	3651.1	3571
9	8460.5	3731.8	3165.3
10	8144.7	3842.7	3275.9
11	7309.1	3431.1	3689.7
12	7445.8	3927.6	3587.5
13	6963.1	3547	3907.5
14	8577.6	3550.5	3140.4
15	8404.2	3733.8	3897.4
Avg.	7623.0	3615.7	3477.6

Table S4 The color rendering index (CRI) of LED (1), LED (2) and LED (3). Each condition was packaged with 15 LEDs.

Num.	LED (1)	LED (2)	LED (3)
1	80.69	75.19	75.24
2	82.93	73.80	73.35
3	80.67	76.43	78.68
4	81.68	75.56	75.58
5	80.86	73.47	78.47
6	82.47	73.92	75.93
7	80.73	74.31	73.96
8	80.86	75.11	73.79
9	82.91	73.23	78.27
10	80.82	75.90	73.44
11	82.81	75.91	78.30
12	80.43	76.86	74.60
13	82.44	73.22	73.90
14	81.19	75.54	78.72
15	80.68	75.23	74.58
Avg.	81.48	74.91	75.79

Table S5 Average of CIE coordinates, CCT and CRI for LED (1), LED (2) and LED (3).

Item	LED (1)	LED (2)	LED (3)
CIE coordinates (x, y)	(0.3017, 0.3012)	(0.4029, 0.3968)	(0.4111, 0.4014)
CCT (K)	7623.0	3615.7	3477.6
CRI	81.48	74.91	75.79
